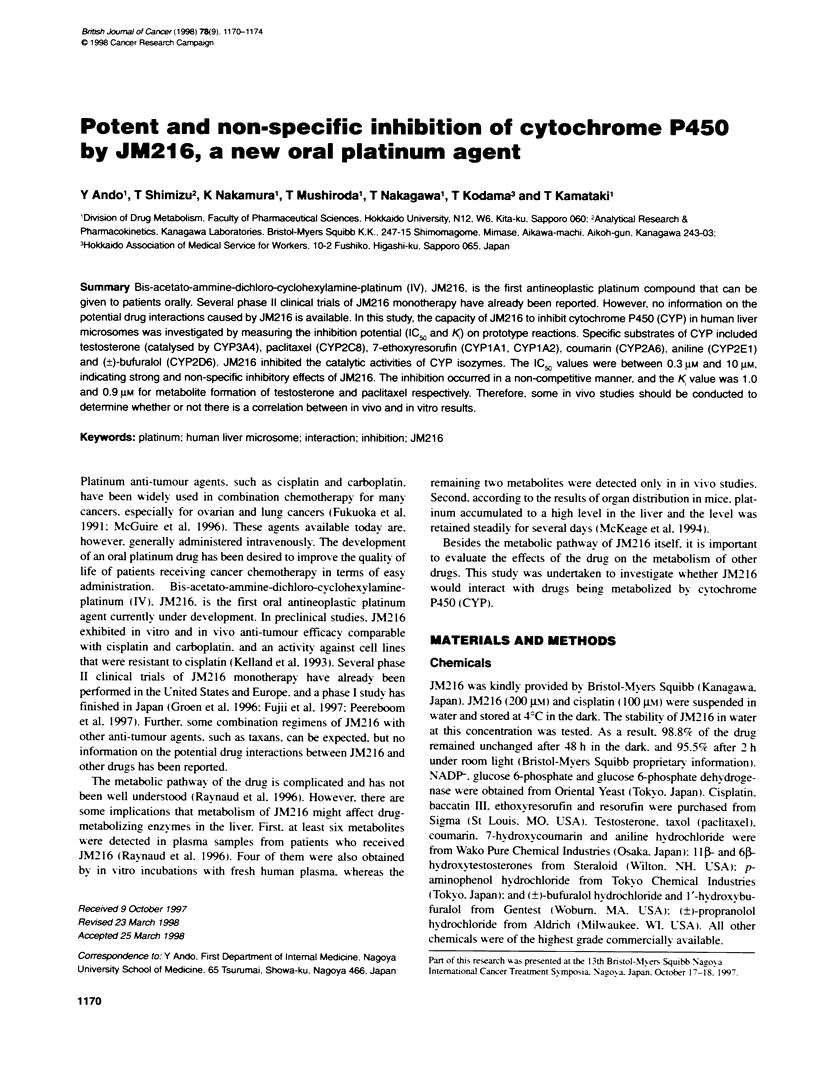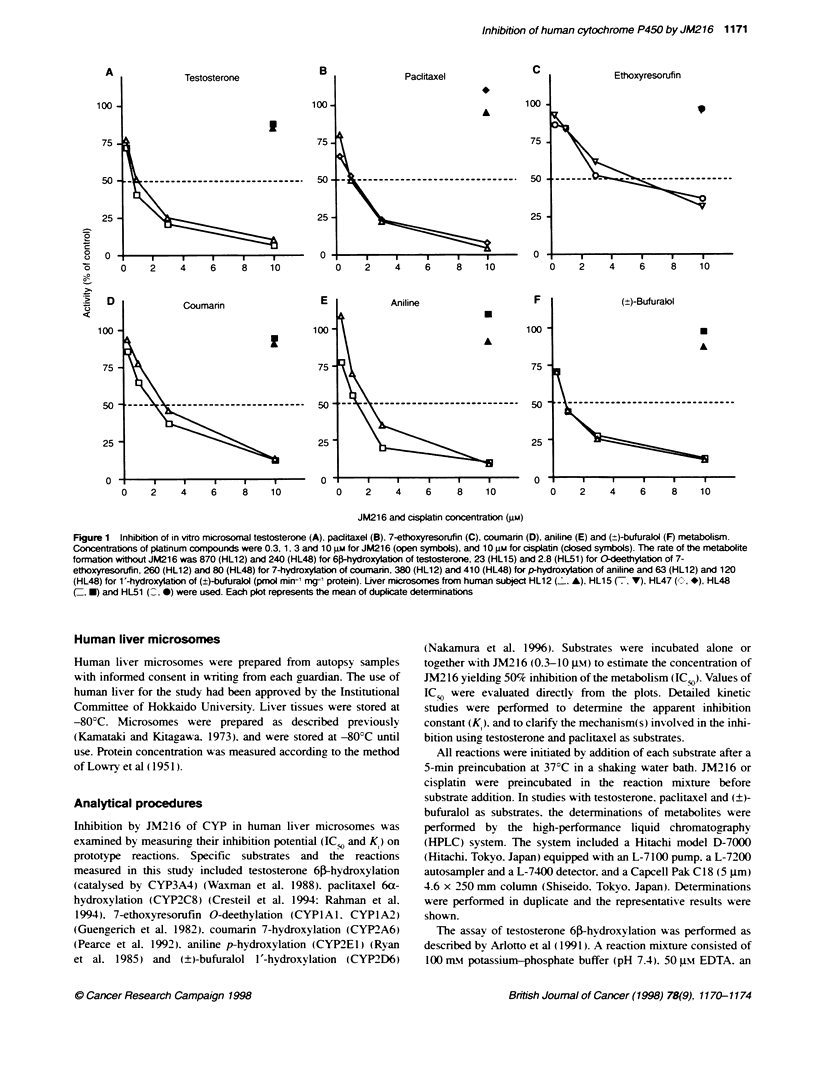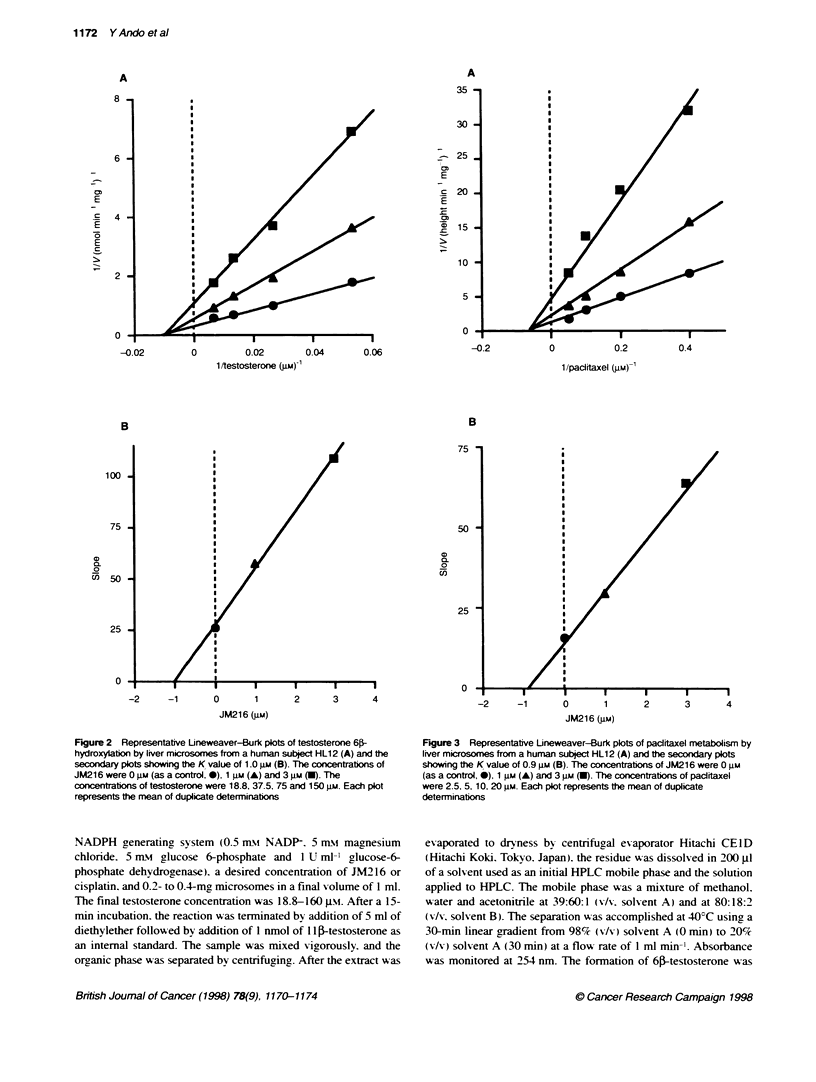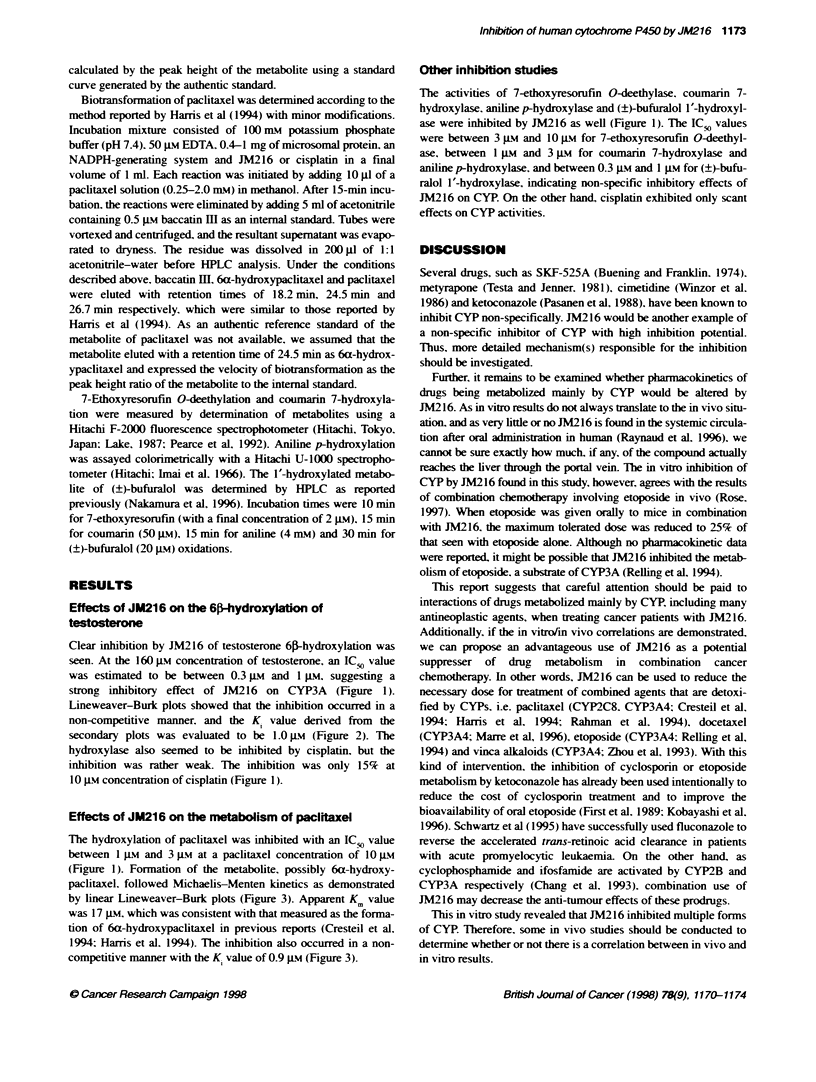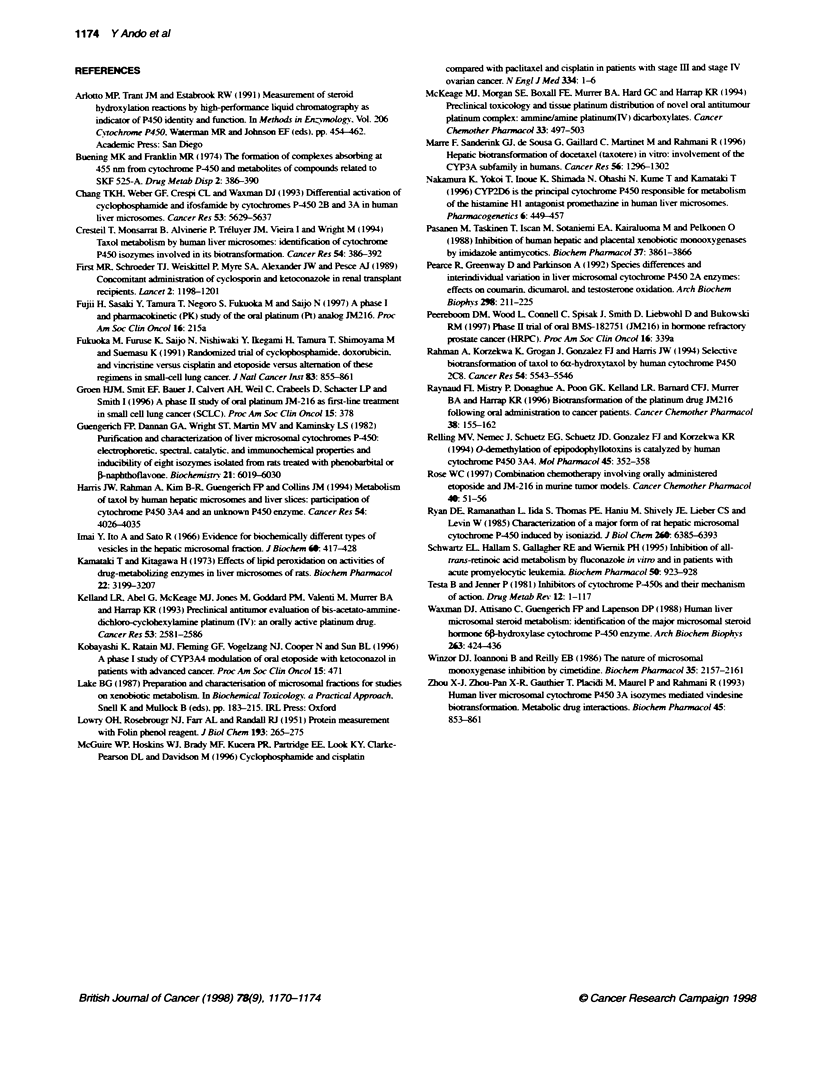# Potent and non-specific inhibition of cytochrome P450 by JM216, a new oral platinum agent.

**DOI:** 10.1038/bjc.1998.649

**Published:** 1998-11

**Authors:** Y. Ando, T. Shimizu, K. Nakamura, T. Mushiroda, T. Nakagawa, T. Kodama, T. Kamataki

**Affiliations:** Division of Drug Metabolism, Faculty of Pharmaceutical Sciences, Hokkaido University, Sapporo, Japan.

## Abstract

Bis-acetato-ammine-dichloro-cyclohexylamine-platinum (IV), JM216, is the first antineoplastic platinum compound that can be given to patients orally. Several phase II clinical trials of JM216 monotherapy have already been reported. However, no information on the potential drug interactions caused by JM216 is available. In this study, the capacity of JM216 to inhibit cytochrome P450 (CYP) in human liver microsomes was investigated by measuring the inhibition potential (IC50 and Ki) on prototype reactions. Specific substrates of CYP included testosterone (catalysed by CYP3A4), paclitaxel (CYP2C8), 7-ethoxyresorufin (CYP1A1, CYP1A2), coumarin (CYP2A6), aniline (CYP2E1) and (+/-)-bufuralol (CYP2D6). JM216 inhibited the catalytic activities of CYP isozymes. The IC50 values were between 0.3 microM and 10 microM, indicating strong and non-specific inhibitory effects of JM216. The inhibition occurred in a non-competitive manner, and the Ki value was 1.0 and 0.9 microM for metabolite formation of testosterone and paclitaxel respectively. Therefore, some in vivo studies should be conducted to determine whether or not there is a correlation between in vivo and in vitro results.


British Journal of Cancer (1998) 78(9), 1170–1174
© 1998 Cancer Research Campaign

### Potent and non-specific inhibition of cytochrome P450 by JM216, a new oral platinum agent

**Y Ando[1], T Shimizu[2], K Nakamura[1], T Mushiroda[1], T Nakagawa[1], T Kodama[3] and T Kamataki[1]**

[1]Division of Drug Metabolism, Faculty of Pharmaceutical Sciences, Hokkaido University, N12, W6, Kita-ku, Sapporo 060; [2]Analytical Research & Pharmacokinetics, Kanagawa Laboratories, Bristol-Myers Squibb K.K., 247-15 Shimomagome, Mimase, Aikawa-machi, Aikoh-gun, Kanagawa 243-03; [3]Hokkaido Association of Medical Service for Workers, 10-2 Fushiko, Higashi-ku, Sapporo 065, Japan

**Summary** Bis-acetato-ammine-dichloro-cyclohexylamine-platinum (IV), JM216, is the first antineoplastic platinum compound that can be given to patients orally. Several phase II clinical trials of JM216 monotherapy have already been reported. However, no information on the potential drug interactions caused by JM216 is available. In this study, the capacity of JM216 to inhibit cytochrome P450 (CYP) in human liver microsomes was investigated by measuring the inhibition potential ($IC_{50}$ and $K_i$) on prototype reactions. Specific substrates of CYP included testosterone (catalysed by CYP3A4), paclitaxel (CYP2C8), 7-ethoxyresorufin (CYP1A1, CYP1A2), coumarin (CYP2A6), aniline (CYP2E1) and (±)-bufuralol (CYP2D6). JM216 inhibited the catalytic activities of CYP isozymes. The $IC_{50}$ values were between 0.3 μM and 10 μM, indicating strong and non-specific inhibitory effects of JM216. The inhibition occurred in a non-competitive manner, and the $K_i$ value was 1.0 and 0.9 μM for metabolite formation of testosterone and paclitaxel respectively. Therefore, some in vivo studies should be conducted to determine whether or not there is a correlation between in vivo and in vitro results.

**Keywords:** platinum; human liver microsome; interaction; inhibition; JM216

Platinum anti-tumour agents, such as cisplatin and carboplatin, have been widely used in combination chemotherapy for many cancers, especially for ovarian and lung cancers (Fukuoka et al, 1991; McGuire et al, 1996). These agents available today are, however, generally administered intravenously. The development of an oral platinum drug has been desired to improve the quality of life of patients receiving cancer chemotherapy in terms of easy administration. Bis-acetato-ammine-dichloro-cyclohexylamine-platinum (IV), JM216, is the first oral antineoplastic platinum agent currently under development. In preclinical studies, JM216 exhibited in vitro and in vivo anti-tumour efficacy comparable with cisplatin and carboplatin, and an activity against cell lines that were resistant to cisplatin (Kelland et al, 1993). Several phase II clinical trials of JM216 monotherapy have already been performed in the United States and Europe, and a phase I study has finished in Japan (Groen et al, 1996; Fujii et al, 1997; Peereboom et al, 1997). Further, some combination regimens of JM216 with other anti-tumour agents, such as taxans, can be expected, but no information on the potential drug interactions between JM216 and other drugs has been reported.

The metabolic pathway of the drug is complicated and has not been well understood (Raynaud et al, 1996). However, there are some implications that metabolism of JM216 might affect drug-metabolizing enzymes in the liver. First, at least six metabolites were detected in plasma samples from patients who received JM216 (Raynaud et al, 1996). Four of them were also obtained by in vitro incubations with fresh human plasma, whereas the remaining two metabolites were detected only in in vivo studies. Second, according to the results of organ distribution in mice, platinum accumulated to a high level in the liver and the level was retained steadily for several days (McKeage et al, 1994).

Besides the metabolic pathway of JM216 itself, it is important to evaluate the effects of the drug on the metabolism of other drugs. This study was undertaken to investigate whether JM216 would interact with drugs being metabolized by cytochrome P450 (CYP).

*Received 9 October 1997*
*Revised 23 March 1998*
*Accepted 25 March 1998*

*Correspondence to:* Y Ando, First Department of Internal Medicine, Nagoya University School of Medicine, 65 Tsurumai, Showa-ku, Nagoya 466, Japan

### MATERIALS AND METHODS

### Chemicals

JM216 was kindly provided by Bristol-Myers Squibb (Kanagawa, Japan). JM216 (200 μM) and cisplatin (100 μM) were suspended in water and stored at 4°C in the dark. The stability of JM216 in water at this concentration was tested. As a result, 98.8% of the drug remained unchanged after 48 h in the dark, and 95.5% after 2 h under room light (Bristol-Myers Squibb proprietary information). $NADP^+$, glucose 6-phosphate and glucose 6-phosphate dehydrogenase were obtained from Oriental Yeast (Tokyo, Japan). Cisplatin, baccatin III, ethoxyresorufin and resorufin were purchased from Sigma (St Louis, MO, USA). Testosterone, taxol (paclitaxel), coumarin, 7-hydroxycoumarin and aniline hydrochloride were from Wako Pure Chemical Industries (Osaka, Japan); 11β- and 6β-hydroxytestosterones from Steraloid (Wilton, NH, USA); *p*-aminophenol hydrochloride from Tokyo Chemical Industries (Tokyo, Japan); and (±)-bufuralol hydrochloride and 1′-hydroxybufuralol from Gentest (Woburn, MA, USA); (±)-propranolol hydrochloride from Aldrich (Milwaukee, WI, USA). All other chemicals were of the highest grade commercially available.

Part of this research was presented at the 13th Bristol-Myers Squibb Nagoya International Cancer Treatment Symposia, Nagoya, Japan, October 17–18, 1997.

**Figure 1** Inhibition of in vitro microsomal testosterone (**A**), paclitaxel (**B**), 7-ethoxyresorufin (**C**), coumarin (**D**), aniline (**E**) and (±)-bufuralol (**F**) metabolism. Concentrations of platinum compounds were 0.3, 1, 3 and 10 μM for JM216 (open symbols), and 10 μM for cisplatin (closed symbols). The rate of the metabolite formation without JM216 was 870 (HL12) and 240 (HL48) for 6β-hydroxylation of testosterone, 23 (HL15) and 2.8 (HL51) for *O*-deethylation of 7-ethoxyresorufin, 260 (HL12) and 80 (HL48) for 7-hydroxylation of coumarin, 380 (HL12) and 410 (HL48) for *p*-hydroxylation of aniline and 63 (HL12) and 120 (HL48) for 1′-hydroxylation of (±)-bufuralol (pmol $min^{-1}$ $mg^{-1}$ protein). Liver microsomes from human subject HL12 (△, ▲), HL15 (▽, ▼), HL47 (◇, ◆), HL48 (□, ■) and HL51 (○, ●) were used. Each plot represents the mean of duplicate determinations

### Human liver microsomes

Human liver microsomes were prepared from autopsy samples with informed consent in writing from each guardian. The use of human liver for the study had been approved by the Institutional Committee of Hokkaido University. Liver tissues were stored at −80°C. Microsomes were prepared as described previously (Kamataki and Kitagawa, 1973), and were stored at −80°C until use. Protein concentration was measured according to the method of Lowry et al (1951).

### Analytical procedures

Inhibition by JM216 of CYP in human liver microsomes was examined by measuring their inhibition potential ($IC_{50}$ and $K_i$) on prototype reactions. Specific substrates and the reactions measured in this study included testosterone 6β-hydroxylation (catalysed by CYP3A4) (Waxman et al, 1988), paclitaxel 6α-hydroxylation (CYP2C8) (Cresteil et al, 1994; Rahman et al, 1994), 7-ethoxyresorufin *O*-deethylation (CYP1A1, CYP1A2) (Guengerich et al, 1982), coumarin 7-hydroxylation (CYP2A6) (Pearce et al, 1992), aniline *p*-hydroxylation (CYP2E1) (Ryan et al, 1985) and (±)-bufuralol 1′-hydroxylation (CYP2D6) (Nakamura et al, 1996). Substrates were incubated alone or together with JM216 (0.3–10 μM) to estimate the concentration of JM216 yielding 50% inhibition of the metabolism ($IC_{50}$). Values of $IC_{50}$ were evaluated directly from the plots. Detailed kinetic studies were performed to determine the apparent inhibition constant ($K_i$), and to clarify the mechanism(s) involved in the inhibition using testosterone and paclitaxel as substrates.

All reactions were initiated by addition of each substrate after a 5-min preincubation at 37°C in a shaking water bath. JM216 or cisplatin were preincubated in the reaction mixture before substrate addition. In studies with testosterone, paclitaxel and (±)-bufuralol as substrates, the determinations of metabolites were performed by the high-performance liquid chromatography (HPLC) system. The system included a Hitachi model D-7000 (Hitachi, Tokyo, Japan) equipped with an L-7100 pump, a L-7200 autosampler and a L-7400 detector, and a Capcell Pak C18 (5 μm) 4.6 × 250 mm column (Shiseido, Tokyo, Japan). Determinations were performed in duplicate and the representative results were shown.

The assay of testosterone 6β-hydroxylation was performed as described by Arlotto et al (1991). A reaction mixture consisted of 100 mM potassium–phosphate buffer (pH 7.4), 50 μM EDTA, an

© Cancer Research Campaign 1998

Figure 2 Representative Lineweaver–Burk plots of testosterone 6β-hydroxylation by liver microsomes from a human subject HL12 (A) and the secondary plots showing the $K_i$ value of 1.0 µM (B). The concentrations of JM216 were 0 µM (as a control, ●), 1 µM (▲) and 3 µM (■). The concentrations of testosterone were 18.8, 37.5, 75 and 150 µM. Each plot represents the mean of duplicate determinations

Figure 3 Representative Lineweaver–Burk plots of paclitaxel metabolism by liver microsomes from a human subject HL12 (A) and the secondary plots showing the $K_i$ value of 0.9 µM (B). The concentrations of JM216 were 0 µM (as a control, ●), 1 µM (▲) and 3 µM (■). The concentrations of paclitaxel were 2.5, 5, 10, 20 µM. Each plot represents the mean of duplicate determinations

NADPH generating system (0.5 mM $NADP^+$, 5 mM magnesium chloride, 5 mM glucose 6-phosphate and 1 U $ml^{-1}$ glucose-6-phosphate dehydrogenase), a desired concentration of JM216 or cisplatin, and 0.2- to 0.4-mg microsomes in a final volume of 1 ml. The final testosterone concentration was 18.8–160 µM. After a 15-min incubation, the reaction was terminated by addition of 5 ml of diethylether followed by addition of 1 nmol of 11β-testosterone as an internal standard. The sample was mixed vigorously, and the organic phase was separated by centrifuging. After the extract was evaporated to dryness by centrifugal evaporator Hitachi CE1D (Hitachi Koki, Tokyo, Japan), the residue was dissolved in 200 µl of a solvent used as an initial HPLC mobile phase and the solution applied to HPLC. The mobile phase was a mixture of methanol, water and acetonitrile at 39:60:1 (v/v, solvent A) and at 80:18:2 (v/v, solvent B). The separation was accomplished at 40°C using a 30-min linear gradient from 98% (v/v) solvent A (0 min) to 20% (v/v) solvent A (30 min) at a flow rate of 1 ml $min^{-1}$. Absorbance was monitored at 254 nm. The formation of 6β-testosterone was

© Cancer Research Campaign 1998

calculated by the peak height of the metabolite using a standard curve generated by the authentic standard.

Biotransformation of paclitaxel was determined according to the method reported by Harris et al (1994) with minor modifications. Incubation mixture consisted of 100 mM potassium phosphate buffer (pH 7.4), 50 μM EDTA, 0.4–1 mg of microsomal protein, an NADPH-generating system and JM216 or cisplatin in a final volume of 1 ml. Each reaction was initiated by adding 10 μl of a paclitaxel solution (0.25–2.0 mM) in methanol. After 15-min incubation, the reactions were eliminated by adding 5 ml of acetonitrile containing 0.5 μM baccatin III as an internal standard. Tubes were vortexed and centrifuged, and the resultant supernatant was evaporated to dryness. The residue was dissolved in 200 μl of 1:1 acetonitrile–water before HPLC analysis. Under the conditions described above, baccatin III, 6α-hydroxypaclitaxel and paclitaxel were eluted with retention times of 18.2 min, 24.5 min and 26.7 min respectively, which were similar to those reported by Harris et al (1994). As an authentic reference standard of the metabolite of paclitaxel was not available, we assumed that the metabolite eluted with a retention time of 24.5 min as 6α-hydroxypaclitaxel and expressed the velocity of biotransformation as the peak height ratio of the metabolite to the internal standard.

7-Ethoxyresorufin *O*-deethylation and coumarin 7-hydroxylation were measured by determination of metabolites using a Hitachi F-2000 fluorescence spectrophotometer (Hitachi, Tokyo, Japan; Lake, 1987; Pearce et al, 1992). Aniline *p*-hydroxylation was assayed colorimetrically with a Hitachi U-1000 spectrophotometer (Hitachi; Imai et al, 1966). The 1′-hydroxylated metabolite of (±)-bufuralol was determined by HPLC as reported previously (Nakamura et al, 1996). Incubation times were 10 min for 7-ethoxyresorufin (with a final concentration of 2 μM), 15 min for coumarin (50 μM), 15 min for aniline (4 mM) and 30 min for (±)-bufuralol (20 μM) oxidations.

### RESULTS

### Effects of JM216 on the 6β-hydroxylation of testosterone

Clear inhibition by JM216 of testosterone 6β-hydroxylation was seen. At the 160 μM concentration of testosterone, an $IC_{50}$ value was estimated to be between 0.3 μM and 1 μM, suggesting a strong inhibitory effect of JM216 on CYP3A (Figure 1). Lineweaver–Burk plots showed that the inhibition occurred in a non-competitive manner, and the $K_i$ value derived from the secondary plots was evaluated to be 1.0 μM (Figure 2). The hydroxylase also seemed to be inhibited by cisplatin, but the inhibition was rather weak. The inhibition was only 15% at 10 μM concentration of cisplatin (Figure 1).

### Effects of JM216 on the metabolism of paclitaxel

The hydroxylation of paclitaxel was inhibited with an $IC_{50}$ value between 1 μM and 3 μM at a paclitaxel concentration of 10 μM (Figure 1). Formation of the metabolite, possibly 6α-hydroxypaclitaxel, followed Michaelis–Menten kinetics as demonstrated by linear Lineweaver–Burk plots (Figure 3). Apparent $K_m$ value was 17 μM, which was consistent with that measured as the formation of 6α-hydroxypaclitaxel in previous reports (Cresteil et al, 1994; Harris et al, 1994). The inhibition also occurred in a non-competitive manner with the $K_i$ value of 0.9 μM (Figure 3).

### Other inhibition studies

The activities of 7-ethoxyresorufin *O*-deethylase, coumarin 7-hydroxylase, aniline *p*-hydroxylase and (±)-bufuralol 1′-hydroxylase were inhibited by JM216 as well (Figure 1). The $IC_{50}$ values were between 3 μM and 10 μM for 7-ethoxyresorufin *O*-deethylase, between 1 μM and 3 μM for coumarin 7-hydroxylase and aniline *p*-hydroxylase, and between 0.3 μM and 1 μM for (±)-bufuralol 1′-hydroxylase, indicating non-specific inhibitory effects of JM216 on CYP. On the other hand, cisplatin exhibited only scant effects on CYP activities.

### DISCUSSION

Several drugs, such as SKF-525A (Buening and Franklin, 1974), metyrapone (Testa and Jenner, 1981), cimetidine (Winzor et al, 1986) and ketoconazole (Pasanen et al, 1988), have been known to inhibit CYP non-specifically. JM216 would be another example of a non-specific inhibitor of CYP with high inhibition potential. Thus, more detailed mechanism(s) responsible for the inhibition should be investigated.

Further, it remains to be examined whether pharmacokinetics of drugs being metabolized mainly by CYP would be altered by JM216. As in vitro results do not always translate to the in vivo situation, and as very little or no JM216 is found in the systemic circulation after oral administration in human (Raynaud et al, 1996), we cannot be sure exactly how much, if any, of the compound actually reaches the liver through the portal vein. The in vitro inhibition of CYP by JM216 found in this study, however, agrees with the results of combination chemotherapy involving etoposide in vivo (Rose, 1997). When etoposide was given orally to mice in combination with JM216, the maximum tolerated dose was reduced to 25% of that seen with etoposide alone. Although no pharmacokinetic data were reported, it might be possible that JM216 inhibited the metabolism of etoposide, a substrate of CYP3A (Relling et al, 1994).

This report suggests that careful attention should be paid to interactions of drugs metabolized mainly by CYP, including many antineoplastic agents, when treating cancer patients with JM216. Additionally, if the in vitro/in vivo correlations are demonstrated, we can propose an advantageous use of JM216 as a potential suppresser of drug metabolism in combination cancer chemotherapy. In other words, JM216 can be used to reduce the necessary dose for treatment of combined agents that are detoxified by CYPs, i.e. paclitaxel (CYP2C8, CYP3A4; Cresteil et al, 1994; Harris et al, 1994; Rahman et al, 1994), docetaxel (CYP3A4; Marre et al, 1996), etoposide (CYP3A4; Relling et al, 1994) and vinca alkaloids (CYP3A4; Zhou et al, 1993). With this kind of intervention, the inhibition of cyclosporin or etoposide metabolism by ketoconazole has already been used intentionally to reduce the cost of cyclosporin treatment and to improve the bioavailability of oral etoposide (First et al, 1989; Kobayashi et al, 1996). Schwartz et al (1995) have successfully used fluconazole to reverse the accelerated *trans*-retinoic acid clearance in patients with acute promyelocytic leukaemia. On the other hand, as cyclophosphamide and ifosfamide are activated by CYP2B and CYP3A respectively (Chang et al, 1993), combination use of JM216 may decrease the anti-tumour effects of these prodrugs.

This in vitro study revealed that JM216 inhibited multiple forms of CYP. Therefore, some in vivo studies should be conducted to determine whether or not there is a correlation between in vivo and in vitro results.

© Cancer Research Campaign 1998

### REFERENCES

Arlotto MP, Trant JM and Estabrook RW (1991) Measurement of steroid hydroxylation reactions by high-performance liquid chromatography as indicator of P450 identity and function. In *Methods in Enzymology*, Vol. 206 *Cytochrome P450*, Waterman MR and Johnson EF (eds), pp. 454–462. Academic Press: San Diego

Buening MK and Franklin MR (1974) The formation of complexes absorbing at 455 nm from cytochrome P-450 and metabolites of compounds related to SKF 525-A. *Drug Metab Disp* **2**: 386–390

Chang TKH, Weber GF, Crespi CL and Waxman DJ (1993) Differential activation of cyclophosphamide and ifosfamide by cytochromes P-450 2B and 3A in human liver microsomes. *Cancer Res* **53**: 5629–5637

Cresteil T, Monsarrat B, Alvinerie P, Tréluyer JM, Vieira I and Wright M (1994) Taxol metabolism by human liver microsomes: identification of cytochrome P450 isozymes involved in its biotransformation. *Cancer Res* **54**: 386–392

First MR, Schroeder TJ, Weiskittel P, Myre SA, Alexander JW and Pesce AJ (1989) Concomitant administration of cyclosporin and ketoconazole in renal transplant recipients. *Lancet* **2**: 1198–1201

Fujii H, Sasaki Y, Tamura T, Negoro S, Fukuoka M and Saijo N (1997) A phase I and pharmacokinetic (PK) study of the oral platinum (Pt) analog JM216. *Proc Am Soc Clin Oncol* **16**: 215a

Fukuoka M, Furuse K, Saijo N, Nishiwaki Y, Ikegami H, Tamura T, Shimoyama M and Suemasu K (1991) Randomized trial of cyclophosphamide, doxorubicin, and vincristine versus cisplatin and etoposide versus alternation of these regimens in small-cell lung cancer. *J Natl Cancer Inst* **83**: 855–861

Groen HJM, Smit EF, Bauer J, Calvert AH, Weil C, Crabeels D, Schacter LP and Smith I (1996) A phase II study of oral platinum JM-216 as first-line treatment in small cell lung cancer (SCLC). *Proc Am Soc Clin Oncol* **15**: 378

Guengerich FP, Dannan GA, Wright ST, Martin MV and Kaminsky LS (1982) Purification and characterization of liver microsomal cytochromes P-450: electrophoretic, spectral, catalytic, and immunochemical properties and inducibility of eight isozymes isolated from rats treated with phenobarbital or β-naphthoflavone. *Biochemistry* **21**: 6019–6030

Harris JW, Rahman A, Kim B-R, Guengerich FP and Collins JM (1994) Metabolism of taxol by human hepatic microsomes and liver slices: participation of cytochrome P450 3A4 and an unknown P450 enzyme. *Cancer Res* **54**: 4026–4035

Imai Y, Ito A and Sato R (1966) Evidence for biochemically different types of vesicles in the hepatic microsomal fraction. *J Biochem* **60**: 417–428

Kamataki T and Kitagawa H (1973) Effects of lipid peroxidation on activities of drug-metabolizing enzymes in liver microsomes of rats. *Biochem Pharmacol* **22**: 3199–3207

Kelland LR, Abel G, McKeage MJ, Jones M, Goddard PM, Valenti M, Murrer BA and Harrap KR (1993) Preclinical antitumor evaluation of bis-acetato-ammine-dichloro-cyclohexylamine platinum (IV): an orally active platinum drug. *Cancer Res* **53**: 2581–2586

Kobayashi K, Ratain MJ, Fleming GF, Vogelzang NJ, Cooper N and Sun BL (1996) A phase I study of CYP3A4 modulation of oral etoposide with ketoconazol in patients with advanced cancer. *Proc Am Soc Clin Oncol* **15**: 471

Lake BG (1987) Preparation and characterisation of microsomal fractions for studies on xenobiotic metabolism. In *Biochemical Toxicology: a Practical Approach*, Snell K and Mullock B (eds), pp. 183–215. IRL Press: Oxford

Lowry OH, Rosebrougr NJ, Farr AL and Randall RJ (1951) Protein measurement with Folin phenol reagent. *J Biol Chem* **193**: 265–275

McGuire WP, Hoskins WJ, Brady MF, Kucera PR, Partridge EE, Look KY, Clarke-Pearson DL and Davidson M (1996) Cyclophosphamide and cisplatin compared with paclitaxel and cisplatin in patients with stage III and stage IV ovarian cancer. *N Engl J Med* **334**: 1–6

McKeage MJ, Morgan SE, Boxall FE, Murrer BA, Hard GC and Harrap KR (1994) Preclinical toxicology and tissue platinum distribution of novel oral antitumour platinum complex: ammine/amine platinum(IV) dicarboxylates. *Cancer Chemother Pharmacol* **33**: 497–503

Marre F, Sanderink GJ, de Sousa G, Gaillard C, Martinet M and Rahmani R (1996) Hepatic biotransformation of docetaxel (taxotere) in vitro: involvement of the CYP3A subfamily in humans. *Cancer Res* **56**: 1296–1302

Nakamura K, Yokoi T, Inoue K, Shimada N, Ohashi N, Kume T and Kamataki T (1996) CYP2D6 is the principal cytochrome P450 responsible for metabolism of the histamine H1 antagonist promethazine in human liver microsomes. *Pharmacogenetics* **6**: 449–457

Pasanen M, Taskinen T, Iscan M, Sotaniemi EA, Kairaluoma M and Pelkonen O (1988) Inhibition of human hepatic and placental xenobiotic monooxygenases by imidazole antimycotics. *Biochem Pharmacol* **37**: 3861–3866

Pearce R, Greenway D and Parkinson A (1992) Species differences and interindividual variation in liver microsomal cytochrome P450 2A enzymes: effects on coumarin, dicumarol, and testosterone oxidation. *Arch Biochem Biophys* **298**: 211–225

Peereboom DM, Wood L, Connell C, Spisak J, Smith D, Liebwohl D and Bukowski RM (1997) Phase II trial of oral BMS-182751 (JM216) in hormone refractory prostate cancer (HRPC). *Proc Am Soc Clin Oncol* **16**: 339a

Rahman A, Korzekwa K, Grogan J, Gonzalez FJ and Harris JW (1994) Selective biotransformation of taxol to 6α-hydroxytaxol by human cytochrome P450 2C8. *Cancer Res* **54**: 5543–5546

Raynaud FI, Mistry P, Donaghue A, Poon GK, Kelland LR, Barnard CFJ, Murrer BA and Harrap KR (1996) Biotransformation of the platinum drug JM216 following oral administration to cancer patients. *Cancer Chemother Pharmacol* **38**: 155–162

Relling MV, Nemec J, Schuetz EG, Schuetz JD, Gonzalez FJ and Korzekwa KR (1994) *O*-demethylation of epipodophyllotoxins is catalyzed by human cytochrome P450 3A4. *Mol Pharmacol* **45**: 352–358

Rose WC (1997) Combination chemotherapy involving orally administered etoposide and JM-216 in murine tumor models. *Cancer Chemother Pharmacol* **40**: 51–56

Ryan DE, Ramanathan L, Iida S, Thomas PE, Haniu M, Shively JE, Lieber CS and Levin W (1985) Characterization of a major form of rat hepatic microsomal cytochrome P-450 induced by isoniazid. *J Biol Chem* **260**: 6385–6393

Schwartz EL, Hallam S, Gallagher RE and Wiernik PH (1995) Inhibition of all-*trans*-retinoic acid metabolism by fluconazole *in vitro* and in patients with acute promyelocytic leukemia. *Biochem Pharmacol* **50**: 923–928

Testa B and Jenner P (1981) Inhibitors of cytochrome P-450s and their mechanism of action. *Drug Metab Rev* **12**: 1–117

Waxman DJ, Attisano C, Guengerich FP and Lapenson DP (1988) Human liver microsomal steroid metabolism: identification of the major microsomal steroid hormone 6β-hydroxylase cytochrome P-450 enzyme. *Arch Biochem Biophys* **263**: 424–436

Winzor DJ, Ioannoni B and Reilly EB (1986) The nature of microsomal monoxygenase inhibition by cimetidine. *Biochem Pharmacol* **35**: 2157–2161

Zhou X-J, Zhou-Pan X-R, Gauthier T, Placidi M, Maurel P and Rahmani R (1993) Human liver microsomal cytochrome P450 3A isozymes mediated vindesine biotransformation. Metabolic drug interactions. *Biochem Pharmacol* **45**: 853–861

© Cancer Research Campaign 1998